# Joseph Furlonge Shekleton

**Published:** 1903-06

**Authors:** 


### JOSEPH FURLONGE SHEKLETON, A.B., M.D.,

*Deputy Surgeon-General.*

The late Deputy Surgeon-General J. F. Shekleton, of the Indian Medical Service (Bombay), had retired for many years from military and civil life in India, but had nevertheless led a very useful life in Clifton, where he was well-known and much respected. He died on April 15th, 1903, at his residence, 11 Victoria Square, Clifton, at the age of 82. It is therefore many years since he was educated at Trinity College, Dublin, where he graduated in both arts and medicine. He also joined the Royal College of Surgeons of Ireland, having become a Fellow in the year 1845. He entered the Army in the same year, and served with the 3rd Troop Bombay Horse Artillery in the Punjab Campaign of 1848–49, and was present during the second siege of Mooltan, including the siege and capture of the town and storming of the citadel. He was also present at the battle of Gujerat, the pursuit of the Sikh army until its final surrender at Rawil Pindi, the occupation of Attock and Peshawur, and the expulsion of the Afghan force beyond the Khyber Pass. For these services he was awarded a medal and two clasps. Subsequently, while in England on sick leave, he qualified for employment in the Assay Department of the Indian Mints, and on his return to duty was appointed Deputy Assay Master at Bombay. After a few months he was transferred to a like position in the Mint at Calcutta, where he became Assay Master, and frequently acted as Master of the Mint and Head Commissioner of Currency. He published, in *The Friend of India*, December 31st, 1868, a most valuable series of assay tables of Indian and other gold and silver coins. These tables contain a large amount of original information regarding Indian coins not previously assayed, and including those of adjoining Asiatic countries.

After his retirement in 1872, Dr. Shekleton settled at Stoke Bishop, near his old friends, Dr. H. H. Goodeve and Dr. Edward Goodeve, and there he took an active part in all parochial questions. He also took a warm interest in the welfare of the Bristol Royal Infirmary, to which he had been appointed a member of the committee at the annual meeting in 1874. After thirteen years of active and useful work as member of committee and house visitor, during which time he introduced many very important reforms and improvements, he resigned his seat at the committee table in 1887, and succeeded Mr. William Trenerry in the office of secretary. It is not yet forgotten how successfully the Institution was controlled for many years by the infusion of military discipline and system; the improvements were great and have been permanent, but in 1895, in consequence of failing health, Dr. Shekleton found it needful to resign his office, and he afterwards led a life of quiet retirement from all active duty. He will be long remembered for his assiduous devotion to the best interests of the Institution for which he laboured.

